# The complete chloroplast genome of *Prunus clarofolia* (Rosaceae), a wild cherry endemic to China

**DOI:** 10.1080/23802359.2021.2016080

**Published:** 2022-01-05

**Authors:** Jianhui Li, Jiawen Yan, Lin Yu, Wenfu Bai, Dongling Nie, Ying Xiong, Sizheng Wu

**Affiliations:** Institute of Economic Botany, Hunan Botanical Garden, Changsha, China

**Keywords:** *Prunus clarofolia*, chloroplast genome, phylogenetic analysis

## Abstract

*Prunus clarofolia* (Schneid.) Yu et Li is a very attractive wild flowering cherry endemic to China. In this study, the complete chloroplast genome of *P. clarofolia* was assembled. The total length of the chloroplast genome was 157,899 bp, containing a pair of inverted repeat regions of 26,393 bp each, separated by a small single-copy region of 19,142 bp, and a large single-copy region of 85,971 bp. The overall GC content of the chloroplast genome was 36.71%. The genome contained 131 genes, including 85 protein-coding genes, 37 tRNA genes, eight rRNA genes, and one pseudogene. Phylogenetic analysis revealed that *P. clarofolia* and *P. pseudocerasus* showed the closest relationship.

*Prunus clarofolia* (Schneid.) Yu et Li (Rosaceae), also named *Cerasus clarofolia*, is a deciduous shrubs or small trees native to China. It is mainly distributed in Anhui, Gansu, Guizhou, Hebei, Henan, Hubei, Hunan, Yunnan, and Zhejiang province, and usually grows in forests or thickets on mountain slopes at an altitude of 800–3600 m (Li and Bartholomew 2003). A good knowledge of genome information and the phylogenetic position of *P. clarofolia* would contribute to the formulation of efficient strategies for its conservation, management, and utilization. Here, we reported the complete chloroplast genome of *P. clarofolia* and ascertained its phylogenetic position.

The fresh leaves of *P. clarofolia* were collected from Tianmen Mountain in Zhangjiajie city, China (29°3′N, 110°28′E). A specimen was deposited at Herbarium (PE), Institute of Botany, CAS Flora of China (https://www.cvh.ac.cn/spms/detail.php?id=e6f99a52, Caifei Zhang, zhangcf@wbgcas.cn) under the voucher number 02247373, and sequenced DNA can be found in the laboratory of Hunan Botanical Garden (voucher specimen: PCHN2020-5, HBG, 113°2′18.44″,28°6′34.54″). Total genomic DNA was extracted by modified CTAB protocol (Doyle and Doyle 1987). The whole genome was sequenced with the Illumina NovaSeq 6000 Sequencing System following the manufacturer’s protocol (Illumina, San Diego, CA). A total of 5.4 Gb clean data were obtained and then assembled using SPAdes version V 3.10.1 (Bankevich et al. 2012). Finally, the genome assemblies were annotated by using CPGAVAS2 (Shi et al. 2019), followed by manual correction.

The chloroplast genome of *P. clarofolia* (accession number: MT747185) was 157,899 bp in length and contained a pair of inverted repeat regions (IRs) of 26,393 bp each, a small single-copy (SSC) region of 19,142 bp, and a large single-copy (LSC) region of 85,971 bp. The overall GC content of the chloroplast genome was 36.71%, and the values of the LSC, SSC, and IR regions were 34.59%, 30.16%, and 42.53%, respectively. The chloroplast genome contained 131 genes, including 85 protein-coding genes, 37 tRNA genes, eight rRNA genes, and one pseudogene.

We used the whole chloroplast genome sequences of 28 *Prunus* species to ascertain the phylogenetic position of *P. clarofolia*. *Pyrus pyrifolia* (NC_015996) was used as the outgroup. The best-fit model generalized time-reversible (GTR) for DNA sequence evolution was selected using jModelTest v2.1.10 (Darriba et al. 2012) based on Akaike’s information criterion. The phylogenetic tree was constructed with 1000 bootstrap replicates under the maximum-likelihood (ML) inference by using RAxML v8.2.10 (Miller et al. 2010). The ML tree revealed that all the species of *Prunus* were clustered into three clades (bootstrap support value 100%), subgenus *Cerasus*, subgenus *Prunus*, and subgenus *Padus* (Figure 1). This result is consistent with the previous studies (Chin et al. 2014; Yan et al. 2020). In the tree, *P. clarofolia* and *P. pseudocerasus* showed the closest relationship.

**Figure 1.**
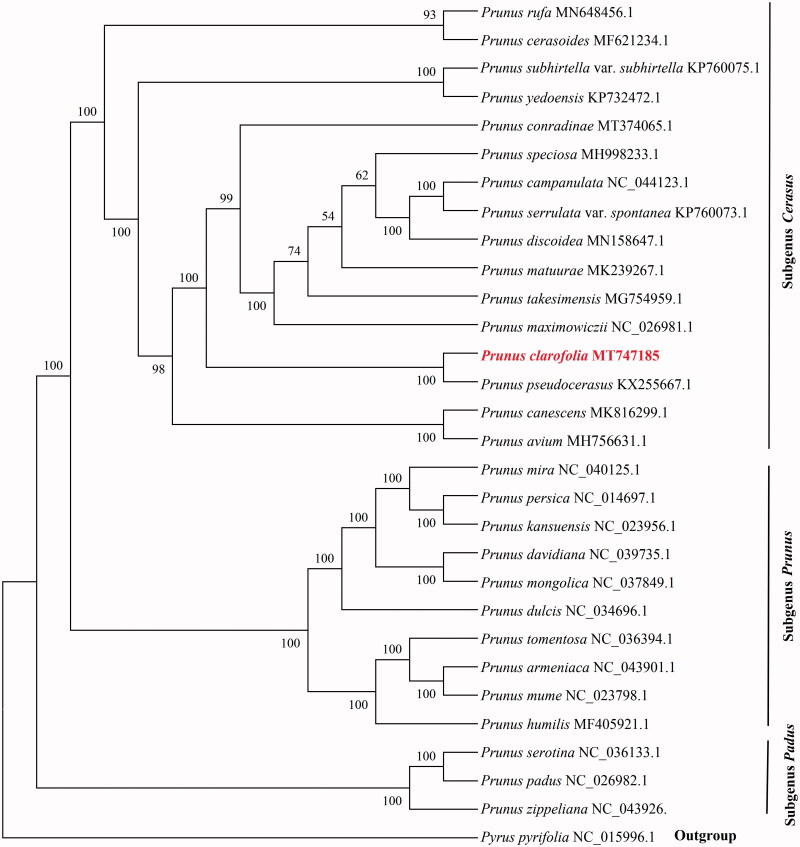
Maximum-likelihood phylogenetic tree of 29 *Prunus* species based on whole chloroplast genome sequence. *Pyrus pyrifolia* (*Pyrus*) was used as an outgroup. The numbers on each node are bootstrap support values.

## Data Availability

The genome sequence data that support the findings of this study are openly available in GenBank of NCBI at https://www.ncbi.nlm.nih.gov under the accession number MT747185. The associated BioProject, SRA, and Bio-Sample numbers are PRJNA739540, SRR14885714, and SAMN19791814, respectively.
